# Enhancing cell death in B-cell malignancies through targeted inhibition of Bcl-3

**DOI:** 10.1038/s41419-024-07067-w

**Published:** 2024-09-26

**Authors:** Renée Daams, Thi Thu Phuong Tran, Mohamed Jemaà, Wondossen Sime, Ruta Mickeviciute, Sara Ek, Lars Rönnstrand, Julhash U. Kazi, Ramin Massoumi

**Affiliations:** 1 Department of Laboratory Medicine, Translational Cancer Research, Lund University, Medicon Village, Lund, Sweden; 2 In Vivo Research Services AB, Scheeletorget 1, Medicon Village, Lund, Sweden; 3 Department of Immunotechnology, Faculty of Engineering, Lund University, Medicon Village, Lund, Sweden; 4 Lund Stem Cell Center, Lund University, Lund, Sweden; 5 Department of Hematology, Oncology and Radiation Physics, Skåne University Hospital, Lund, Sweden

**Keywords:** Leukaemia, Lymphoma

## Abstract

The t(14;19)(q32;q13) is a rare recurring translocation found in B-cell lymphoproliferative malignancies, involving the Bcl-3 gene. This chromosomal translocation is often found in patients under the age of 50 and causes a more progressive disease. The Bcl-3 gene encodes a protein belonging to the IκB family of proteins, which tightly regulates NFκB signaling by acting as an activator or repressor of transcription. Previously, we developed a second-generation Bcl-3 inhibitor that could directly interfere with Bcl-3 signaling pathway, resulting in reduced melanoma cell proliferation, invasion, and migration. The present study aimed to investigate the effect of a Bcl-3 inhibitor on B-cell lymphoma and leukemia cells. It was found that treatment of cells with this inhibitor caused a decrease in cell proliferation and cell survival. Furthermore, Bcl-3 inhibition in B-cell malignant cells resulted in the loss of mitochondrial membrane potential and functionality, as well as the increased expression of cleaved caspase 3, indicating that cell death occurs through the intrinsic apoptotic pathway. This observation is further supported by reduced expression of cIAP1 protein 1 (cIAP1) upon treatment of cancer cells. Given the current lack of clinical advancements targeting Bcl-3 in oncology, this opens a novel avenue for the development and investigation of highly specific therapeutic interventions against B-cell malignancies.

## Introduction

The hematopoietic tree is a highly hierarchical system resulting in the development of myeloid or lymphoid cells [1]. However, aberrant cell signalling can cause the development and progression of several haematological malignancies, including leukemia and lymphoma. The mechanisms that maintain a normal balance between differentiating and activating B-cells are frequently affected in B-cell lymphomas, resulting in a heterogeneous group of cancers [2, 3]. In Sweden lymphoma is the 7th most common type of cancer in both men and women with a median age at diagnosis of 72 years and the relative 5-year survival is approximately 75% [4]. Genetic alterations are frequently occurring events in lymphomagenesis resulting in chromosomal translocations that often involve one of the Ig loci and an oncogene causing cellular abnormalities such as increased cell proliferation, decreased cell death, aberrant differentiation, and cell growth [5]. Recurrent reciprocal translocations are commonly seen in B-cell lymphoma that often involve the IgH loci and one prominent translocation occurring in early B-lymphocyte development involves *IGH* and *BCL2* (t(14;18)(q32;q21)) [6]. Furthermore, other translocations observed in various types of B-cell lymphomas are t(11;14)(q13;q32) between *CCDN1* and *IGH*, causing deregulated cyclin D1 expression and aberrant cell cycle functions. The t(8;14)(q24;q32) translocation involves *IGH* and *MYC* is a common hallmark seen in Burkitt’s lymphoma [7, 8]. Besides chromosomal translocations, gain- and loss-of-function mutations affecting various cellular pathways, such as Nuclear Factor kappa B (NFκB), phosphatidylinositol-3 kinase (PI3K), and JAK signalling pathways result in aberrant B-cell signalling causing dysregulated cell growth and survival [9].

The NFκB family of transcription factors consists of five family members: RelA (p65), RelB, c-Rel, p105/p50, and p100/p52 that can interact with each other as homo- or heterodimers. They all share a common domain called the Rel homology domain, which is necessary for dimerization, interactions with inhibitors of NFκB (IκB) proteins, and DNA binding [10]. NFκB signalling is tightly regulated through the inhibitory function of IκB proteins and IκB kinase (IKK) proteins that phosphorylate and cause subsequent degradation of IκB proteins [11]. One major regulator of the NFκB signalling pathway is the proto-oncogene B-cell lymphoma 3 (Bcl-3), which is an atypical IκB protein [10, 12, 13]. Bcl-3 protein contains ankyrin repeat domains and transactivation domains, essential for its interaction with NFκB proteins p50 and p52, to which Bcl-3 selectively binds. [14–16]. Bcl-3 was originally identified as a recurrent translocation t(14;19) in chronic lymphocytic leukemia (CLL) leading to upregulation of Bcl-3 contributing to disease progression [17]. Bcl-3 translocation has been associated with a more progressive disease, the development of secondary abnormalities, and relatively short survival [18, 19]. There has also been found an association between trisomy 12 and t(14;19) Bcl-3 translocation in chronic lymphocytic leukemia patients, indicating that the translocation could be an early event in developing B-cell malignancies [20].

Since the initial discovery of Bcl-3’s involvement in hematological malignancies, subsequent research has revealed its dysregulation in various other cancer types, including colorectal cancer [21, 22], ovarian cancer [23], breast cancer [24–26], cylindromatosis [27], skin cancer [28–30], liver cancer [31], and prostate cancer [32]. The tumorigenic properties of Bcl-3 affect most cellular processes, including cell proliferation [30, 31, 33], cell migration and invasion [24, 26, 34], cell survival [35, 36], as well as promotion of immune escape in cancer through the induction of PD-L1 expression [23]. Even though the NFκB signalling pathway is a relevant therapeutic target with multiple drugs and small molecules used in the clinic or being studied in clinical trials [37, 38]. Recently, a small molecule targeting Bcl-3 demonstrated encouraging outcomes in breast cancer, effectively inhibiting tumor growth and metastasis [39]. In our previous study, high-throughput screening and in vitro experiments identified a lead molecule that could interfere with Bcl-3-mediated cyclin D1 expression and cell proliferation [40]. The molecule A27 was created through the optimization of a lead molecule and chemical engineering. This molecule exhibited substantial interference and binding with Bcl-3 and prevented its downstream signalling pathways. The use of A27 not only hindered cell proliferation, migration, and invasion but also led to a reduction in tumor growth in animal models in vivo *(An optimized Bcl-3 inhibitor for melanoma treatment, Saamarthy* et. al*.)*.

In the present study, our objective was to examine the efficacy of our previously developed Bcl-3 inhibitor in B-cell leukemia and lymphoma. The results revealed that A27 induces apoptosis in B-cell malignant cells. Additionally, treatment of hematological cancer cells with A27 led to cell death through the upregulation of cleaved caspase 3, thereby promoting the intrinsic apoptosis pathway.

## Materials and methods

### Cell culture and reagents

B-cell lymphoma cell lines Karpas-422, SU-DHL-8 and WSU-NHL and B-cell leukemia cell lines RS4;11 and SUP-B15 were cultured in RPMI-1640 supplemented with 20% fetal bovine serum (Biosera) and 100 U/ml penicillin/streptomycin (Corning). B-cell lymphoma cell lines Granta-519, RL and Rec-1 were cultured in RPMI-1640 medium supplemented with 10% fetal bovine serum (Biosera) and 100 U/ml penicillin/streptomycin (Corning). RS4;11 and SUP-B15, and cells were obtained from Deutsche Sammlung von Mikroorganismen und Zellkulturen (DSMZ, Braunschweig, Germany). All cell lines were cultured at 37 °C in a humidified atmosphere containing 5% CO_2_. To confirm the knockdown of Bcl-3 with the pool of small interfering RNA (siRNA) in comparison to control siRNA, RL and Rec-1 cells (2 × 10^5^/well) were transiently transfected with ON-TARGETplus SMARTpool of siRNA targeting BCL-3 (25 nM, Dharmacon, L-003874-00-0005), or ON-TARGETplus Non-targeting Pool (siControl) (25 nM, Dharmacon, D-001810-10-05) using DharmaFECT Transfection reagent (Dharmacon).

ON-TARGETplus SMARTpool of siRNA targeting BCL3, target sequences (4X):

5´-AGACACGCCUCUCCAUAUU-3´

5´-GGCCGGAGGCGCUUUACUA-3´

5´-GCGCAAAUGUACUCCGGCA-3´

5´-GCCGGGAGCUCGACAUCUA-3´

ON-TARGETplus Non-targeting Pool (Control SiRNA), target sequences:

5´-UGGUUUACAUGUCGACUAA-3´

5´-UGGUUUACAUGUUGUGUGA-3´

5´-UGGUUUACAUGUUUUCUGA-3´

5´-UGGUUUACAUGUUUUCCUA-3´

### Cell morphology analysis

Cells were seeded at a cell density of 4 × 10^3^ cells per well on 96-well plates and incubated overnight. Following incubation, cells were treated with DMSO as control or 20 μM A27 and incubated for 48 h at 37 °C and 5% CO_2_. Cell morphology was assessed by brightfield microscopy using the Zeiss inverted microscope.

### Western blot

Cells were harvested by spinning down the cell suspension at lower speed (222 x g) for 5 min. The cells were washed twice in cold PBS and spun down between each wash, at 222 x g for 5 min. Cells were lysed for 30 min in RIPA buffer (150 mM NaCl, 50 mM Tris-HCl pH 7.6, 5 mM EDTA pH 8.0, 0.1% SDS, 1 g sodium deoxycholate, 1% Triton-X-100) supplemented with 40 µl/ml complete protease inhibitors (Roche) and vortexed every third minute. Lysates were cleared through centrifugation in a pre-cooled centrifuge at maximum speed (19280 x *g*) for 10 min at 4 °C and the protein content was determined by Bradford analysis. Equal amounts of protein were electrophoretically separated on 10% gels and transferred onto polyvinylidene difluoride (PVDF) membranes (Thermo Scientific). Membranes were blocked with PBS-T containing 5% non-fat milk or TBS-T containing 5% BSA. Following blocking, membranes were probed with antibodies towards Bcl-3 (SC-185, Santa Cruz), p50 (SC-8414, Santa Cruz), p52 (SC-7386, Santa Cruz), β-Actin (#ab6276, Abcam), cIAP1 (SC-271419, Santa Cruz), or cleaved caspase 3 (Asp175, #9660, Cell Signaling). After incubation with primary antibodies, membranes were probed with polyclonal goat anti-rabbit immunoglobulins/HRP or polyclonal rabbit anti-mouse immunoglobulins/HRP (Dako Denmark A/S). The chemiluminescence was detected with Amersham Imager 600 (GE Healthcare).

### RNA isolation and quantitative real-time PCR

Total RNA was extracted using the RNeasy® Mini kit (Qiagen) following the manufacturer’s instructions. RNA concentrations were measured using a NanoDrop 2000 spectrophotometer (Thermo Scientific). The cDNA synthesis was performed using 10X RT random primers, dNTP mix, 10X RT buffer and Multiscribe Reverse Transcriptase enzyme (Applied Biosystems). The amplifications were run on a T Gradient Thermoblock PCR Thermocycler (Biometra). Real-time detection of the PCR product was performed using SYBR® Green PCR Master Mix (Applied Biosystems). RT-qPCR was run using Quantstudio 7 Flex (Applied Biosystems). All the reactions were performed in triplicates.

The following primer sequences (TAG Copenhagen) were used:

GAPDH sense 5´-TGCACCACCAACTGCTTAGC-3´

GAPDH anti-sense 5´-GGCATGGACTGTGGTCATGAG-3´

Bcl-3 sense 5´-TATTGCTGTGGTGCAGGGTA-3´

Bcl-3 anti-sense 5’-CCACAGACGGTAATGTGGTG-3´

CYCLIN D1 sense 5´- GGCGGAGGAGAACAAACAGA-3´

CYCLIN D1 anti-sense 5´-TGGCACAAGAGGCAACGA-3´

p50 sense 5´-CCCAGTGAAGACCACCTCTC-3´

p50 anti-sense 5´-CTGAGTTTGCGGAAGGATGT-3´

p52 sense 5´-GGACTGGTAGGGGCTGTAGG-3´

p52 anti-sense 5´-CACATGGGTGGAGGCTCT-3´

### Half-maximal inhibitory concentration (IC_50_) determination

To determine the IC_50_ values the following B-cell leukemia cell lines were used: Karpas-422, RS4;11, SU-DHL-8, Rec-1, Granta-519, RL, SUP-B15, and WSU-NHL. Cells were seeded in quadruplicates on a 96-well plate and incubated for 48 h without any treatment (control) or with increasing concentrations of A27 (0.0001 –50 μM). Cell viability and proliferation were measured using WST-1 (Roche) analysis. WST-1 assay measures the number of viable cells with the principle of the reduction of tetrazolium salts (WST-1[2-(4-Iodophenyl)-3-(4-nitrophenyl)-5-(2,4-disulfophenyl)-2H-tetrazolium]) to colored formazon dye by cellular enzymes derived from metabolically active cells. In brief, after 48 h of drug incubation, 10 μl of WST-1 was added to each well and incubated for 3 h at 37 °C before being analyzed using a Synergy 2 plate reader (Biotek).

## Cell proliferation and cell survival assays

### Cell counting

On 24-well plates an equal number of Karpas-422, RS4;11, SUP-B15, SU-DHL-8, and WSU-NHL cells were seeded in triplicates. Following seeding, cells were treated with DMSO (control) or 20 μM A27 and incubated for 0 h, 24 h, 48 h, 72 h, and 96 h at 37 °C and 5% CO_2_. For each time point cells and PBS from washing the wells were collected and centrifuged for 5 min at the speed of 300 x *g*. Cell suspensions were resuspended in a complete medium and cells were manually counted after staining with trypan blue (Thermo Fisher Scientific) using a hemacytometer.

### WST-1 and alamarBlue assay

Cell proliferation was measured using the WST-1 (Roche) or AlamarBlue assay (Thermo Fisher). In brief, an equal number of cells (Karpas-422, RS4;11, SUP-B15, SU-DHL-8, Granta-519, Rec1, RL, and WSU-NHL) were seeded on 96-well flat-bottom plates in 100 μl of complete cell culture medium for 3 h before drug treatments. Cells were treated with DMSO (control) or 20 μM of A27 and incubated for 0 h, 24 h, 48 h, 72 h, and 96 h. After 0–96 h of incubation, 10 μl of WST-1 reagent was added to each well and plates were incubated for 3 h at 37 °C. Absorbance was measured at dual wavelengths of 450 nm and 630 nm using the Synergy 2 HT Multi-Mode Microplate Reader (Biotek). WST-1 is a commonly used assay to measure the number of viable cells with the principle of the reduction of tetrazolium salts (WST-1[2-(4-Iodophenyl)-3-(4-nitrophenyl)-5-(2,4-disulfophenyl)-2H-tetrazolium]) to colored formazon dye by cellular enzymes derived from metabolically active cells. To determine the effect of Bcl-3 knockdown on RL and Rec-1 cell growth, cell proliferation was measured using alamarBlue assay (Thermo Fisher). In brief, RL and Rec-1 cells (10 × 10^3^) were seeded on 96-well flat-bottom plates in 100 μl of the respective complete cell culture medium. After overnight incubation, cells were transiently transfected with the pool of siRNA (control-siRNA or BCL3-siRNA). After 0–96 h of incubation, 10 μl of alamarBlue reagent was added to each well and plates were incubated for 2 h at 37 °C. The changes in the fluorescence of the resazurin dye were measured at 535/35 nm excitation and 590/35 emission wavelengths using the Synergy 2 HT Multi-Mode Microplate Reader (Biotek).

### Cell cycle

Cells were seeded and treated with DMSO, A27 or transiently transfected with the pool of siRNA (control-siRNA or BCL3-siRNA) and incubated for 48 h. Following incubation, cells were collected and washed with PBS and centrifuged for 5 min at the speed of 2971 x *g* before as much PBS as possible was removed from the cell pellet. Cell pellets were resuspended in 500 μl PBS before dropwise being added to 4.5 ml of ice cold 70% ethanol, while carefully being vortexed. Cells were fixed at −20 °C overnight. Samples were centrifuged for 5 min at the speed of 1880 x *g* and ethanol was removed, followed by samples being washed 2x with PBS. After washing, cells are resuspended in PBS containing 20 μg/ml propidium iodide (Sigma Aldrich), 200 μg/ml RNase, and 0.1% Triton-x-100. Cells were incubated for 30 min at 37 °C, followed by cell cycle analysis using FACS. Appropriate controls were used as gating controls for all experiments. All flow cytometric analyses were performed on FACSVerse™ (BD Biosciences). Data were analyzed using FCS Express 6 Flow Research Edition (De Novo software).

### Flow cytometry – Cell death analysis

When indicated WSU-NHL, SUP-B15, Rec-1, and RL cells were seeded and treated with DMSO, A27 or transiently transfected with the pool of siRNA (control-siRNA or BCL3-siRNA) for 48 h. Following incubation with treatment, cells were collected and washed 2x with PBS + 1% BSA (Sigma Aldrich) before one of the following staining protocols was performed:

i. For Annexin V and 7-AAD staining, the FITC Annexin V Apoptosis Detection Kit with 7-AAD (Biolegend) was used following the manufacturer’s instructions.
ii. For the simultaneous quantification of plasma membrane integrity and mitochondrial transmembrane potential (Δψm), cells were harvested and collected with the culture medium and stained with 1 μg/mL propidium iodide (Sigma Aldrich) and 40 nM 3,3’-dihexyloxacarbocyanine iodide (DiOC6 [3] (Invitrogen) for 30 min at 37 °C before FACS assessment.
iii. For staining of mitochondria, cells were labeled for 45 min at 37 °C with 100 nM of the MitoTracker red (Thermo Fisher) before FACS assessment. The signal shift is measured compared to non-treated cells.

Appropriate controls were used as gating controls for all experiments. All flow cytometric analyses were performed on FACSVerse™ (BD Biosciences). Data were analyzed using FCS Express 6 Flow Research Edition (De Novo software).

### GSEA analysis

The transcriptomic data for BB-ALL was sourced from the Therapeutically Applicable Research to Generate Effective Treatments (TARGET) dataset. TARGET is an initiative by the National Cancer Institute (NCI) that aims to collect extensive molecular characterization data across numerous childhood cancers. For DLBCL, the data was obtained from the Gene Expression Omnibus (GEO) series GSE136971. The samples were sorted based on Bcl-3 expression levels. This stratification allowed for a focused analysis of how varying levels of Bcl-3 expression affect the disease’s transcriptomic profile. The top 50 and bottom 50 samples representing the highest and lowest Bcl-3 expression levels, respectively, were selected for further analysis. This approach ensures a stark contrast in Bcl-3 expression, facilitating a more robust comparative analysis. Gene Set Enrichment Analysis (GSEA version 4.3.2, developed by the Broad Institute) was employed for the study. GSEA is a computational method that determines whether a predefined set of genes shows statistically significant differences in expression between two biological states. In this case, it was used to compare the high and low Bcl-3 expression samples. The analysis utilized the Human Collection Molecular Signatures Databases (MSigDBs 2023.2). MSigDB is a collection of annotated gene sets for use with GSEA software. We used the Hallmark gene sets and oncogenic signature gene sets for this analysis. The enrichment was determined in the Bcl-3 high population.

### Statistics

The data shown are expressed as mean ± S.E.M. All experiments requiring statistical analysis were performed at least three times. Statistical analyses were performed using GraphPad Prism Software 9 (GraphPad), and *p* values were calculated using one-way ANOVA and Tukey multiple comparison test, two-way ANOVA and Šidák multiple comparison test, or two-tailed *t-test*. The *p* values are indicated as non-significant (ns) *p ≤ 0.0347, **p ≤ 0.0086, ***p ≤ 0.0008, and ****p ≤ 0.0001.

## Results

### Bcl-3 expression differs among B-cell leukemia and B-cell lymphoma cell lines

**Fig. 1:**
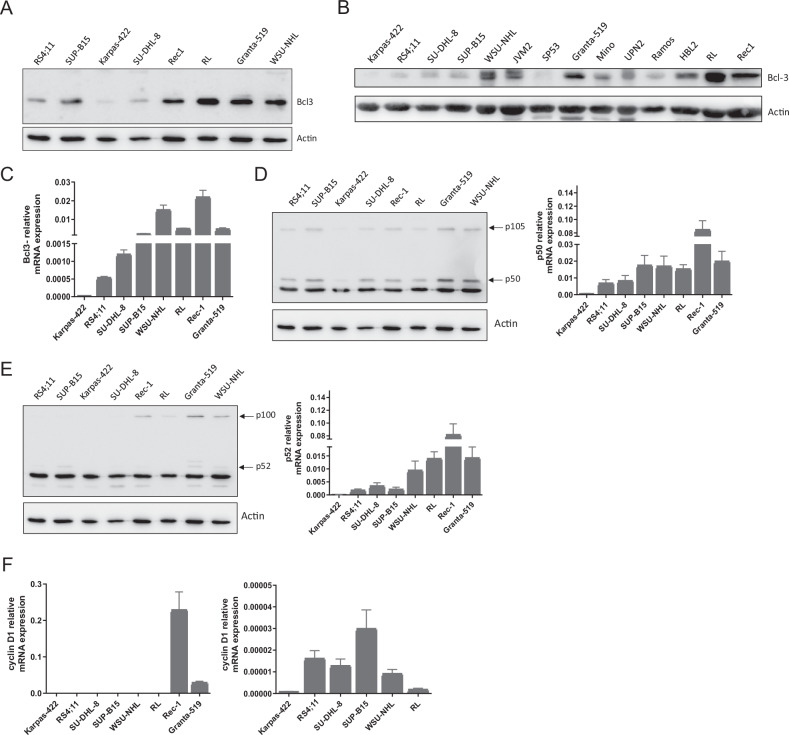
Bcl-3 expression differs among B-cell malignant cell lines. **A** Lysates were prepared from RS4;11, SUP-B15, Karpas-422, SU-DHL-8, Rec-1, RL, Granta-519, and WSU-NHL cells and subjected to Western Blot analysis toward Bcl-3 and Actin. **B** Lysates from a large panel of B-cell leukemia and B-cell lymphoma cells including Karpas-422, RS4;11, SU-DHL-8, WSU-NHL, JVM2, SP53, Granta-519, Mino, UPN2, Ramos, HBL2, RL, and Rec-1 were subjected to Western Blot analysis toward Bcl-3 and Actin. **C** mRNA from Karpas-422, RS4;11, SU-DHL-8, SUP-B15, WSU-NHL, RL, Rec-1, and Granta-519 cells were prepared and subjected to real-time quantitative (qPCR) analysis of Bcl-3 and housekeeping gene GAPDH. All reactions were performed in triplicates. Data are represented as relative mRNA levels mean ± SEM. **D** Left. Lysates from Karpas-422, RS4;11, SU-DHL-8, SUP-B15, WSU-NHL, RL, Rec-1, and Granta-519 cells were subjected to Western Blot analysis toward p105/p50 and Actin Right. mRNA from Karpas-422, RS4;11, SU-DHL-8, SUP-B15, WSU-NHL, RL, Rec-1, and Granta-519 cells were prepared and subjected to real-time quantitative (qPCR) analysis of p50 and housekeeping gene GAPDH. All reactions were performed in triplicates. Data are represented as relative mRNA levels mean ± SEM. **E** Left: Lysates from Karpas-422, RS4;11, SU-DHL-8, SUP-B15, WSU-NHL, RL, Rec-1, and Granta-519 cells were subjected to Western Blot analysis toward p100/p52 and Actin Right: mRNA from Karpas-422, RS4;11, SU-DHL-8, SUP-B15, WSU-NHL, RL, Rec-1, and Granta-519 cells were prepared and subjected to real-time quantitative (qPCR) analysis of p52 and housekeeping gene GAPDH. All reactions were performed in triplicates. Data are represented as relative mRNA levels mean ± SEM. **F** Left: mRNA from Rec-1, and Granta-519 cells were prepared and subjected to real-time quantitative (qPCR) analysis of cyclin D1 and housekeeping gene GAPDH. All reactions were performed in triplicates. Data are represented as relative mRNA levels mean ± SEM. Right: mRNA from Karpas-422, RS4;11, SU-DHL-8, SUP-B15, WSU-NHL, and RL cells were prepared and subjected to real-time quantitative (qPCR) analysis of cyclin D1 and housekeeping gene GAPDH. All reactions were performed in triplicates. Data are represented as relative mRNA levels mean ± SEM.

**Fig. 2:**
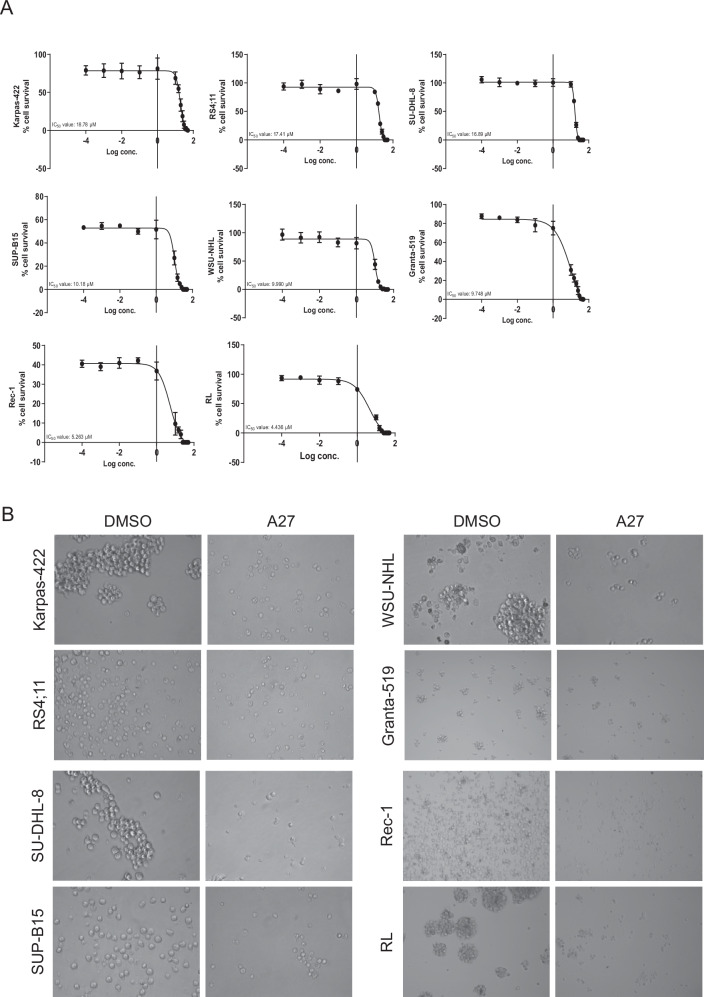
Analyzing the half-maximal inhibitory concentration (IC50) of A27 in B-Cell malignant cell Lines. **A** Karpas-422, RS4;11, SU-DHL-8, SUP-B15, WSU-NHL, RL, Rec-1, and Granta-519 cells were treated with increasing concentrations of A27 for 48 h, followed by determination of the half-maximal inhibitory concentration (IC_50_) using a WST-1 assay. All reactions were performed in quadruplicates. Data are represented as mean ± SEM. **B** Representative morphology images of Karpas-422, RS4;11, SU-DHL-8, SUP-B15, WSU-NHL, RL, Rec-1, and Granta-519 cells after 48 h of treatment with DMSO or A27 (IC_50_ concentration).

Initially, we investigated the expression levels of Bcl-3 in a panel of B-cell leukemia and B-cell lymphoma cell lines (Table 1). Bcl-3 was highly expressed in B-cell lymphoma cell lines: Granta-519, RL, Rec-1, and WSU-NHL, but comparatively low expressed in B-cell lymphoma cell lines: Karpas-422 and SU-DHL-8. B-cell leukemia cell lines RS4;11 and SUP-B15 also showed the high expression of Bcl-3 (Fig. 1A). Upon Bcl-3 protein expression analysis in a larger panel of B-cell lymphoma cell lines, we could confirm that B-cell leukemia cell lines RS4;11 and SUP-B15 express lower levels of Bcl-3 compared to most B-cell lymphoma cell lines (Fig. 1B). Among this large panel of cell lines, RL, Rec-1, Granta-519, WSU-NHL, and JVM2 expressed the highest levels of Bcl-3 (Fig. 1B). Analysis using RT-qPCR established that Karpas-422 expresses the lowest mRNA levels, whereas RL, Rec-1, Granta-519, and WSU-NHL cells express the highest levels of Bcl-3 mRNA (Fig. 1C). It is well-known that Bcl-3 binds to p50 and p52 homodimers to exert its stimulatory and repressive function on target gene transcription [15]. In the cell lines that were tested, expression of p50 and p52 RNA and protein could be detected (Fig. 1D, E). As expected, Rec-1 and Granta-519 exhibited significantly higher levels of cyclin D1 expression compared to the remaining cell lines in the panel because of *t(11:14)* translocation (Fig. 1F). Next, B-cell lymphoma and B-cell leukemia cell lines were treated with increasing concentrations of A27 to establish an IC_50_ value for each cell line (Fig. 2A). In all cell lines, the IC_50_ value was below 20 μM and concentrations above this resulted in 50–100% of cell death. Furthermore, we found that Karpas-422 has the highest IC_50_ value of 18.78 μM, meanwhile, RL has the lowest IC_50_ value of 4.436 μM (Fig. 2A). To investigate whether treatment with A27 can affect cell morphology, cancer cells were cultured in the presence of A27 (IC_50_) for 48 h. Control/DMSO-treated cells showed a typical round morphology without any apparent signs of apoptosis (Fig. 2B). Upon treatment with A27, these cells no longer formed clusters, and a significant number of cells appeared to undergo cell death. Additionally, A27-treated cells were often smaller compared to control-treated cells (Fig. 2B).

**Table 1:** B-cell leukemia and B-cell lymphoma cell lines used in the study and related information concerning the disease, patient age.

Cell line	Cell type	Disease	Patient and age	Mutations and Karyotype
Karpas-422	B-cell lymphoma	Diffuse large B-cell lymphoma	Female, 73	FASR TP53
RS4;11	Lymphoblast	Acute lymphoblastic leukemia	Female, 32	FASLG t(4;11)(q21;q23) Isochromosome 7
SU-DHL-8	B-lymphocyte	Diffuse large B-cell lymphoma	Male, 59	MYC FASR TP53
SUP-B15	B-lymphoblast	Acute lymphoblastic leukemia	Male, 8	46, XY t(9;22)(q34;q11) t(4;14) (p11;q24)
WSU-NHL	B cell lymphoma	Diffuse large B-cell lymphoma	Female, 48	RBBP6 TP53 45, XX t(14;18) (q32;q21)
RL	B-lymphoblast	Non-Hodgkin’s lymphoma	Male, 52	14;18 chromosomal translocation
JVM2	Lymphoblast	Mantle cell lymphoma	Female, 63	TP53 t(11;14)(q13;q32)
DOHH2	B cell lymphoma	Non-Hodgkin’s lymphoma	Male, 60	14;18 Chromosomal translocation BCL-2 major rearrangement
SP53	B cell lymphoma	Mantle cell lymphoma	Female, 58	**--**
Ramos	B lymphocyte	Burkitt’s lymphoma	Male, 3	**--**
Rec-1	B lymphoblast	Mantle cell lymphoma	Male, 57	TP53 t(11;14)(q13;q32)
BJAB	B cell lymphoma	Burkitt’s lymphoma	Female, 5	--
Granta	B cell lymphoma	Mantle cell lymphoma	Female, 58	8% polyploidy t(11;14)
SC-1	B lymphocyte	Follicular lymphoma	Male, 67	
Mino	B lymphoblast	Mantle cell lymphoma	Male, 64	TP53, Cyclin D2 and cyclin D3 overexpression
HBL-2	B cell lymphoma	Mantle cell lymphoma	Male, 84	--
UPN-2	B cell lymphoma	Mantle cell lymphoma	Male, 57	TP53

### B-cell malignant cell proliferation is inhibited by treatment with A27

**Fig. 3:**
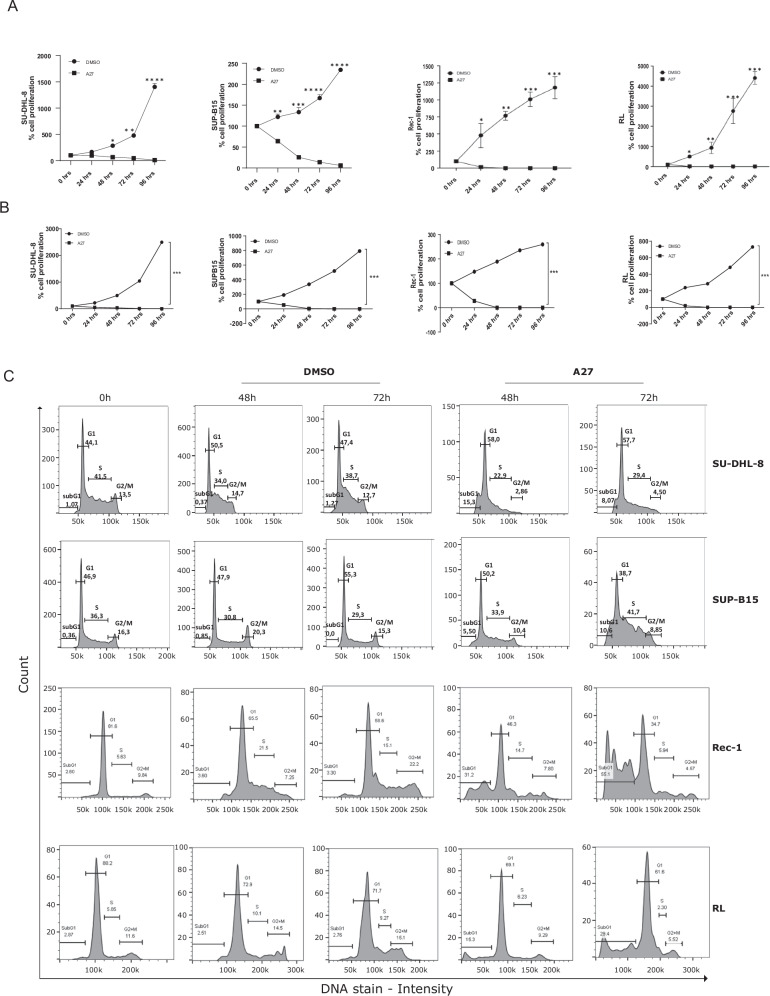
Cell proliferation is inhibited by treatment with A27. **A** SU-DHL-8, SUP-B15, RL, and Rec-1 cells were seeded followed by treatment with DMSO or 20 µM A27 for 0–96 h. Cell proliferation was measured by counting cells manually at 0–96 h. All data are presented as mean ± SEM from three independent experiments. **B** SU-DHL-8, SUP-B15, RL, and Rec-1 cells were treated with 20 µM A27 or DMSO as a control for 0–96 h. After treatment the cells were subjected to WST-1 assay to measure cell proliferation. All data are presented as mean ± SEM from three independent experiments. **C** The cells were treated with DMSO or A27 for 48–72 h before being fixed and stained with propidium iodide. Cell cycle analysis was performed by using flow cytometry. Representative histograms show cell cycle profiles. All data is presented as mean ± SEM from three independent experiments.

To examine the impact of A27 treatment on cell proliferation and survival, we selected SU-DHL-8 and SUP-B15 cell lines, which express moderate levels of Bcl-3, along with RL and Rec-1 cell lines, which exhibit the highest Bcl-3 expression. Cell proliferation was assessed by manually counting the number of cells after treatment with A27 or DMSO. Remarkably, between 24–48 h of A27 treatment, there was a significant reduction in cell proliferation compared to the control treatment (Fig. 3A). The confirmation of this result was achieved through the execution of the WST-1 assay, which involved treating leukemia and lymphoma cells with A27 for a duration of 24 to 96 h (Fig. 3B). The administration of A27 led to an impact on the cell cycle, specifically by increasing the number of cells in the subG1 phase and reducing cyclin D1 promoter activity (Fig. 3C and Supplementary Fig. 1A).

### B-cell malignant cell apoptosis is promoted by treatment with A27

**Fig. 4:**
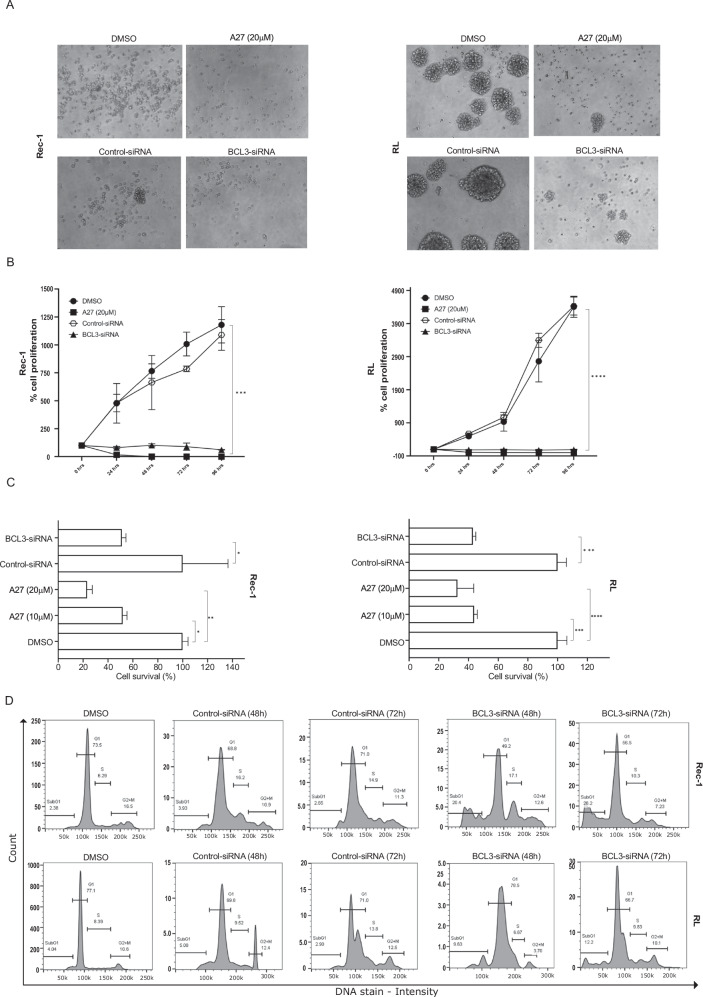
Bcl-3 knockdown reduces cell viability in B-cell lymphoma. **A** Representative morphology images of Rec-1, and RL cells after 48 h of either DMSO and A27 (20 μM) treatment or transiently transfected with the pool of control-siRNA and BCL3-siRNA. **B** Line graphs showing the cell proliferation (%) for Rec-1 and RL cells following 0–96 h of treatment with DMSO, A27 (20 μM), or transiently transfected with the pool of control-siRNA and BCL3-siRNAs. After 0–96 h, cells were subjected to alamarBlue assay to measure cell proliferation. **C** Bar graphs showing the percentages of cell survival after 48 h of treatment with DMSO, A27 (10 μM and 20 μM), or transiently transfected with the pool of control-siRNA and BCL3-siRNA. After 48 h, cells were subjected to alamarBlue assay to measure cell viability and the results were normalized to 100% based on the viability readout recorded from the respective control groups (DMSO and Control-siRNA). **D** Representative histograms showing the cell cycle profile for Rec-1 (Top) and RL (Bottom) cells. Cells were transiently transfected with the pool of control-siRNA and BCL3-siRNAs for 48 and 72 h before being fixed and stained with propidium iodide. Cell cycle analysis was performed by using flow cytometry. All data is presented as mean ± SEM from two independent experiments.

To determine if downregulation of the Bcl-3 by knockdown produces the same phenotype as using A27, we assessed the effects of Bcl-3 siRNA on cell proliferation and cell cycle. Transfection of cells with BCL3-specific siRNA (ON-TARGETplus SMARTpool siRNA) confirmed the downregulation of Bcl-3 (Supplementary Fig. 1B). Subsequently, the cells were examined for morphology, survival, proliferation, and cell cycle analysis. Bcl-3 knockdown cells exhibited similar characteristics to those treated with the A27, including rounded morphology, decreased proliferation rate, increased cell death, and accumulation in the subG1 phase of the cell cycle, compared to the control-siRNA cells (Fig. 4A–D).

**Fig. 5:**
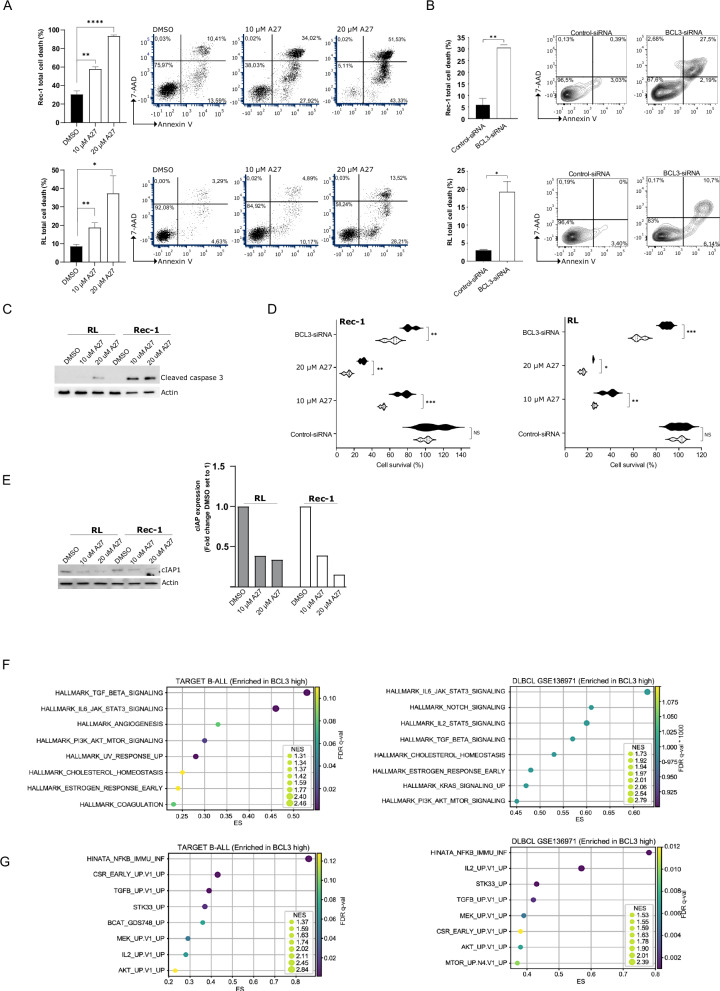
A27 treatment or BCL3 knockdown induced caspase-dependent cell death. Flow cytometry analysis of cell death in Rec-1, and RL cells after treatment with DMSO or A27 as depicted in dot plots (**A**) or transiently transfected with the pool of control-siRNA and BCL3-siRNA as illustrated in contour plots **(B)** for 48 h. Cells were stained with Annexin V and 7-AAD before analysis. Representative dot plots or contour plots show cell survival staining (lower left quadrant, live cells; lower right quadrant, early apoptosis; upper right quadrant, late apoptosis; upper left quadrant, necrosis) of each cell line. Graphs depict mean ± SEM for each cell line from two to three independent experiments. **C** Lysates from RL and Rec-1 cells after 48 h of treatment with DMSO, 10 μM A27, or 20 μM A27 were subjected to Western Blot analysis toward cleaved Caspase 3 and Actin. **D** Violin plots showing the effect of Z-VAD-FMK on reduced cell viability in Rec-1 and RL cells induced by A27 treatment (10 μM and 20 μM) or BCL3-knockdown. The percentages of cell survival were measured with alamarBlue assay after 96 h of Z-VAD-FMK co-treatment (20 μM) with A27 (10 μM and 20 μM) or transiently transfected with the pool of control-siRNA and BCL3-siRNA. The results were normalized to 100% based on the viability readout recorded from the control (control-siRNA) without Z-VAD-FMK treatment. Black: + ZVAD, white: - ZVAD **(E)** Lysates from RL and Rec-1 cells after 48 h of treatment with DMSO, 10 μM A27, or 20 μM A27 were subjected to Western Blot analysis toward cIAP and Actin. Bar graph depicts the quantification of cleaved caspase 3 expression levels normalized against DMSO. **F**, **G** Gene set enrichment analysis comparing BCL3 high vs BCL3 low samples. The figure presents the enrichment of distinct Hallmark gene sets and Oncogenic signatures in samples exhibiting elevated BCL3 expression within the B-ALL (TARGET dataset) and DLBCL (GSE136971 dataset). Enrichment scores (ES) and normalized enrichment scores (NES) are provided for each gene set and pathway, indicating their relative expression levels in samples with higher BCL3 expression. All pathways provided here passed the *p*-value cut-off of 0.05.

Toward investigating the impact of A27 on programmed cell death, we subjected Rec-1 and RL cells to a 48 h treatment with either DMSO or A27 as well as treatment with Bcl-3 siRNA or control siRNA. Thereafter, the cells were stained with Annexin V and 7-AAD followed by fluorescence-activated cell sorting (FACS) analysis. These experiments showed a significant increase in cell death after treatment with A27 and Bcl-3 siRNA compared to control-treated cells (Fig. 5A, B). Furthermore, analysis of cleaved caspase 3 levels, showed an increase in cleaved caspase 3 upon treatment with A27 compared to DMSO-treated cells (Fig. 5C). To investigate if the cell death induced by A27- and Bcl-3 siRNA could be inhibited by a caspase inhibitor, cells were treated with Z-VAD-FMK. This treatment significantly increased cell viability in A27- and Bcl-3 siRNA-transfected cells compared to control cells (Fig. 5D). Additionally, we could show a reduction in pro-survival gene cellular inhibitor of apoptosis 1 (cIAP1) expression following treatment with A27 (Fig. 5E). Taken together, treatment with A27 elevates cell death as seen by morphological changes, cell viability, apoptosis, and caspase 3 cleavage. To understand how Bcl-3 expression affects overall cellular signaling in B-cell malignancies, we analyzed mRNA expression data from DLBCL and B-ALL patients. We performed Gene Set Enrichment Analysis (GSEA) by categorizing the patients into groups based on high and low levels of Bcl-3 expression. This analysis revealed a distinct enrichment of specific Hallmark gene sets and Oncogenic signatures when comparing high and low Bcl-3 expression in B-ALL and DLBCL. In samples exhibiting elevated Bcl-3 expression (Fig. 5F–G), there was a notable enrichment of Transforming growth factor-beta (TGF-β), Interleukin 6 (IL-6), Phosphoinositide 3-kinases (PI3K), and NFκB immune response signaling pathways, ranking among the top pathways associated with high Bcl-3 expression levels (Fig. 5F, G).

### A27 activates the intrinsic apoptotic pathway in B-cell malignant cell lines

**Fig. 6:**
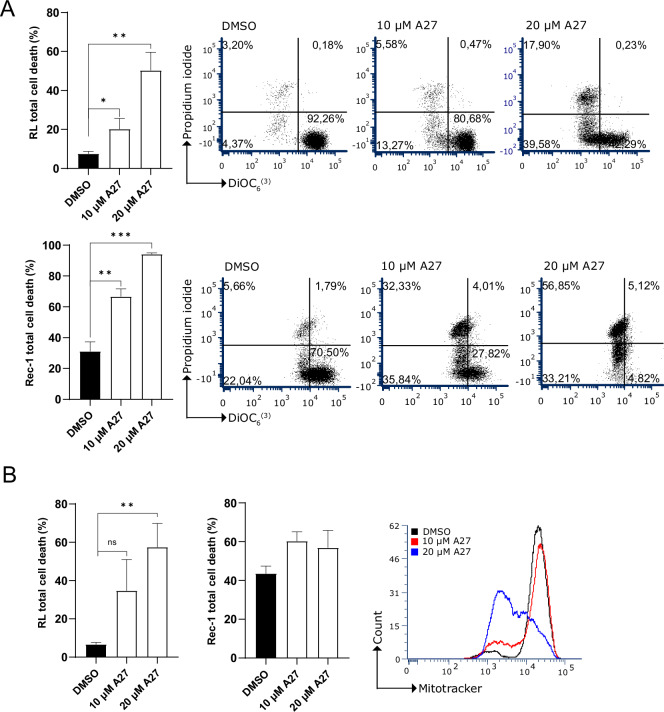
A27 induces cell death by activating the intrinsic apoptotic pathway. **A**, **B** Flow cytometry analysis of, RL, and Rec-1 cells after 48-hour treatment with DMSO control or A27, followed by staining with propidium iodide and DiOC_6_[3]. Representative dot plots show cell survival staining (lower right quadrant, live cells; lower left quadrant, early apoptosis; upper left quadrant, late apoptosis; upper right quadrant, necrosis) of each cell line. Graphs depict mean ± SEM for each cell line from three independent experiments. **C** Flow cytometry analysis of RL and Rec-1 cells after 48-hour treatment with DMSO control or A27, followed by staining with MitoTracker Red dye. Data are presented as representative overlay histograms for DMSO (black line), 10 μM A27 (red line), and 20 μM A27 (blue line). Graphs depict mean ± SEM for each cell line from three independent experiments. mutations, and karyotype.

To find whether the mitochondrial membrane potential (∆ψm) is affected by treating cells with A27, Rec-1, and RL cells were co-stained with propidium iodide to check plasma membrane integrity and DiOC_6_ [3] to measure mitochondria membrane potential. Treatment with A27 significantly increased ∆ψm and mitochondria membrane permeability compared to the control cells (Fig. 6A). Furthermore, upon treating cells with A27 and subsequently staining with MitoTracker to assess mitochondrial membrane potential and functionality, RL and Rec-1 cells demonstrated a loss of MitoTracker Red dye, indicating a decline in mitochondrial membrane potential (∆ψm, Fig. 6B). Taken together, our data suggests that treatment with A27 causes increased cell death by potentially activating the intrinsic apoptotic signaling pathway, as shown by the loss of mitochondrial membrane potential and functionality.

## Discussion

Non-Hodgkin lymphoma (NHL) is the most common hematological malignancy worldwide, responsible for 3.3% of all cancer-related deaths [41]. In Sweden, NHL is the seventh most common type of cancer, with a relative 5-year survival rate of 76.4% and a relative 10-year survival rate of 65.4% [4]. NHL is a heterogeneous disease originating from B- and T-cell populations. This disease comprises different subtypes based on epidemiology, genetic alterations, clinical features, and therapy strategies [42]. Treatment of lymphoma depends on the symptoms of the patients, type, stage, and histology, but the current standard of care with a cure rate of 60% is R-CHOP (rituximab, cyclophosphamide, doxorubicin, vincristine, and prednisone) chemotherapy [43]. However, even though most patients go into remission following standard-of-care treatment, 20–40% of all patients will subsequently relapse, most of them during the first 2-3 years after completion of therapy [44, 45]. Since lymphoma is often diagnosed at late stages, with a lack of curative treatments and a relatively high relapse rate, there is an urgent need for novel treatment therapies.

Most B-cell malignancies are the result of infections, chronic inflammation, or chromosomal translocations. A recurring translocation frequently observed in B-cell lymphomas is t(14;19)(q32;q13), which involves the Bcl-3 gene. This translocation results in increased Bcl-3 expression, leading to a more aggressive disease with a relatively shorter survival rate [18, 19]. Additionally, Bcl-3 is involved in the development and progression of several solid types of cancer (For review see [46]). Previously, our research group has developed a small molecule inhibitor against Bcl-3 that could directly interfere with Bcl-3-mediated cyclin D1 activity in melanoma resulting in decreased cell proliferation, cell migration, and invasion, as well as decreased tumor growth in vivo [40]. In the present study, we aimed to expand our findings by investigating the effects of A27 on B-cell malignancies.

Initially, we determined the expression levels of Bcl-3 in a large panel of B-cell malignant cell lines and found that Bcl-3 was highly expressed in certain types of these cells. In samples from patients with B-ALL and DLBCL, we also detected heightened levels of Bcl-3 expression along with the enrichment of various pathways, such as NFκB immune response PI3K/AKT/mTOR, IL-6, and TGF-β signaling pathways. These pathways, commonly dysregulated in cancer, were notably more pronounced in individuals exhibiting elevated Bcl-3 expression compared to lower levels of Bcl-3. As Bcl-3 preferentially binds to p50 and p52 transcription factors to activate or repress gene transcription [15], the expression of p50 and p52 was intact in the cells harboring high levels of Bcl-3. Following IC_50_ calculations, it was detected that Granta-519, Rec-1, and RL cells were more sensitive, while the highest IC_50_ value was calculated for Karpas-422 cells. Indeed, Karpas-422 cells express the lowest levels of Bcl-3. In addition to IC_50_, A27 caused a significant reduction in cell proliferation in all cell lines compared to control-treated cells. This effect was confirmed by cell survival assay and manual cell counting. As A27-treated cells were dying, we performed FACS analysis after staining cells with Annexin V and 7-AAD to measure cell membrane scrambling, phosphatidylserine translocation, and chromatin condensation. Treatment with A27 caused an increase in Annexin V and 7-AAD uptake, confirming that B-cell malignant cells underwent apoptosis. Previous report could show that Bcl-3 expression inhibits DNA damage-induced p53 activation, thereby suppressing p53-induced apoptosis by inducing Hdm2 [35]. In prostate cancer, inhibitor of DNA-binding (Id) proteins 1 and 2 were reduced upon Bcl-3 knock-down, leading to a sensitization of chemotherapeutic drug-induced apoptosis [32]. Furthermore, in breast cancer Bcl-3 stabilizes CtBP1, causing a decrease in CtBP1-dependent pro-apoptotic genes *p21* and *NOXA* [47]. Our data, in conjunction with the earlier published studies, suggest that Bcl-3 is an anti-apoptotic marker that can protect cells from undergoing programmed cell death in B-cell malignant cells.

Moreover, our observations indicate that A27 induces cell death, potentially by activating the intrinsic apoptotic pathway, which leads to increased permeability of the mitochondria membrane and reduced mitochondrial membrane potential (∆ψm). Analysis of the expression levels of cleaved caspase 3 and cIAP1 revealed that A27 promotes caspase 3 cleavage while decreasing the expression of cIAP1. Collectively, the treatment of B-cell malignant cells with A27 results in cell death, as evidenced by morphological changes such as cell shrinkage and blebbing, augmented expression of Annexin V and 7-AAD, loss of mitochondrial membrane potential (∆ψm) as indicated by DiOC6 [3] and MitoTracker staining, elevated levels of caspase 3, and decreased levels of cIAP1. In cutaneous T-cell Lymphoma knockdown of Bcl-3 decreases the expression of cIAP1 and cIAP2, resulting in reduced cell survival [48]. Furthermore, activated Bcl-3 deficient T-cells caused abnormal rapid cell death of T-cells due to activated Bim, indicating that Bcl-3 is a vital pro-survival gene for T-cells [49].

Many efforts have been addressed to create targeted therapies against the NFκB signaling pathway due to its involvement in many types of cancer [38]. Recently, reduced cell migration was observed upon inhibition of pirin with a small molecule that could dissolve the interaction between pirin and Bcl-3 [50]. In the present study, we investigated the effect of Bcl-3 antagonist A27 on B-cell lymphoma and leukemia. We could provide evidence that A27 treatment causes reduced cell proliferation and increased cell death. Future studies should aim at performing transcriptome sequencing to identify changes in gene expression patterns associated with NFκB activity in particular p50 and p52 upon A27 administration as well as exploring the tissue distribution and uptake of this compound to gain a comprehensive understanding of its pharmacokinetics and potential effects on different organs or systems.

## Supplementary information

Suppl. Fig. 1

Original WB data

## Data Availability

The datasets generated and analysed during the current study are available from the corresponding author on reasonable request. The western blots generated or analysed during this study are included in this published article as supplementary information files.
