# Supplementary material for: Enhancing cell death in B-cell malignancies through targeted inhibition of Bcl-3
Source: Cell Death Dis. 2024 Sep 26;15(9):690. doi: 10.1038/s41419-024-07067-w (PMC11427694; doi:10.1038/s41419-024-07067-w)
Supplement: Supplementary file 1 — Suppl. Fig. 1 [file 41419_2024_7067_MOESM1_ESM.pdf]

Supplementary Figure 1

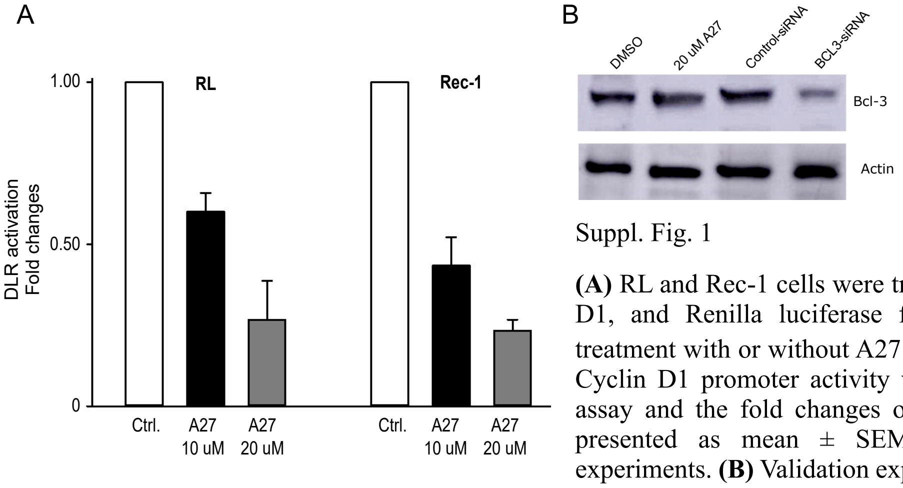

Suppl. Fig. 1

(A) RL and Rec-1 cells were transfected with Bcl-3, cyclin D1, and Renilla luciferase for 24 hours, followed by treatment with or without A27 (10 or 20 µM) for 24 hours. Cyclin D1 promoter activity was measured by luciferase assay and the fold changes over the control (DMSO) is presented as mean ± SEM from three independent experiments. **(B)** Validation experiment testing the levels of Bcl-3 knockdown by transiently transfecting cells with the pool of Control-siRNA and BCL3-siRNA for 48 hours before lysing and wester blotting.
