# Supplementary figures and images for: Enhancing cell death in B-cell malignancies through targeted inhibition of Bcl-3
Source: Cell Death Dis. 2024 Sep 26;15(9):690. doi: 10.1038/s41419-024-07067-w (PMC11427694; doi:10.1038/s41419-024-07067-w)

Fig. 1A

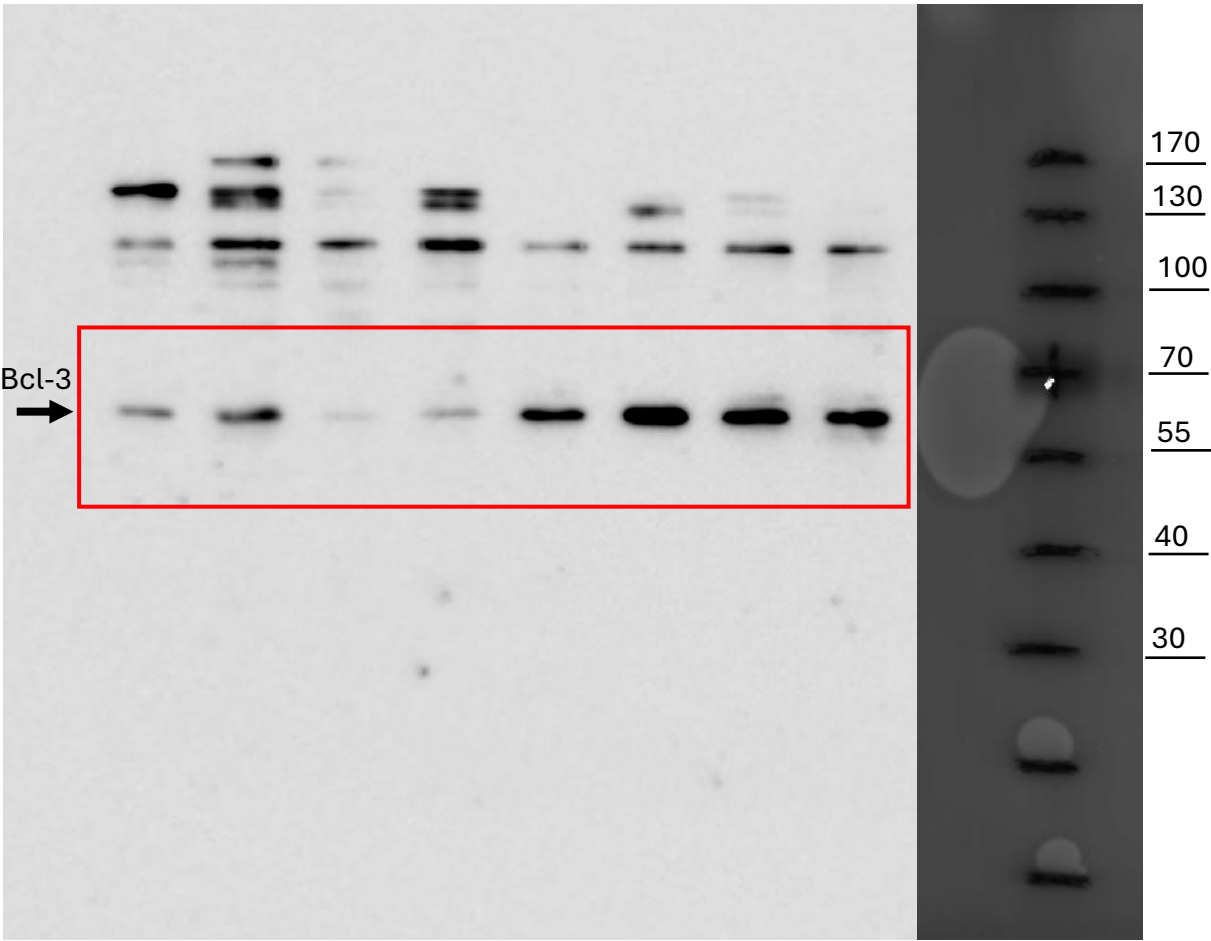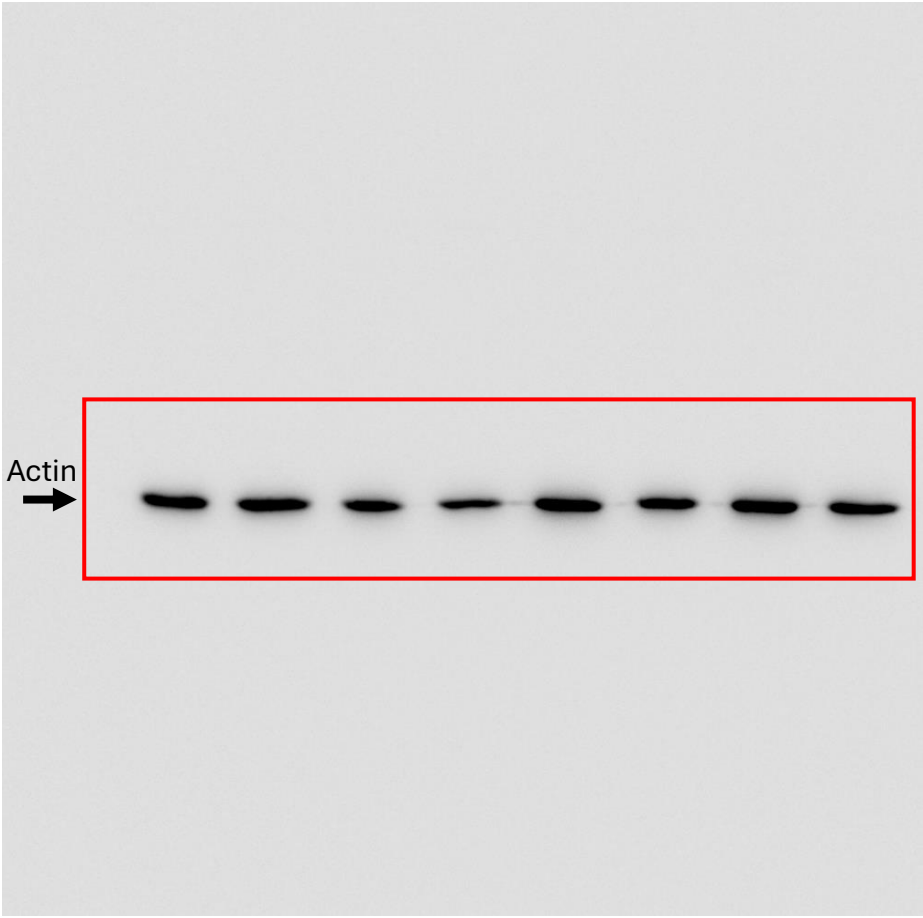

Fig. 1B

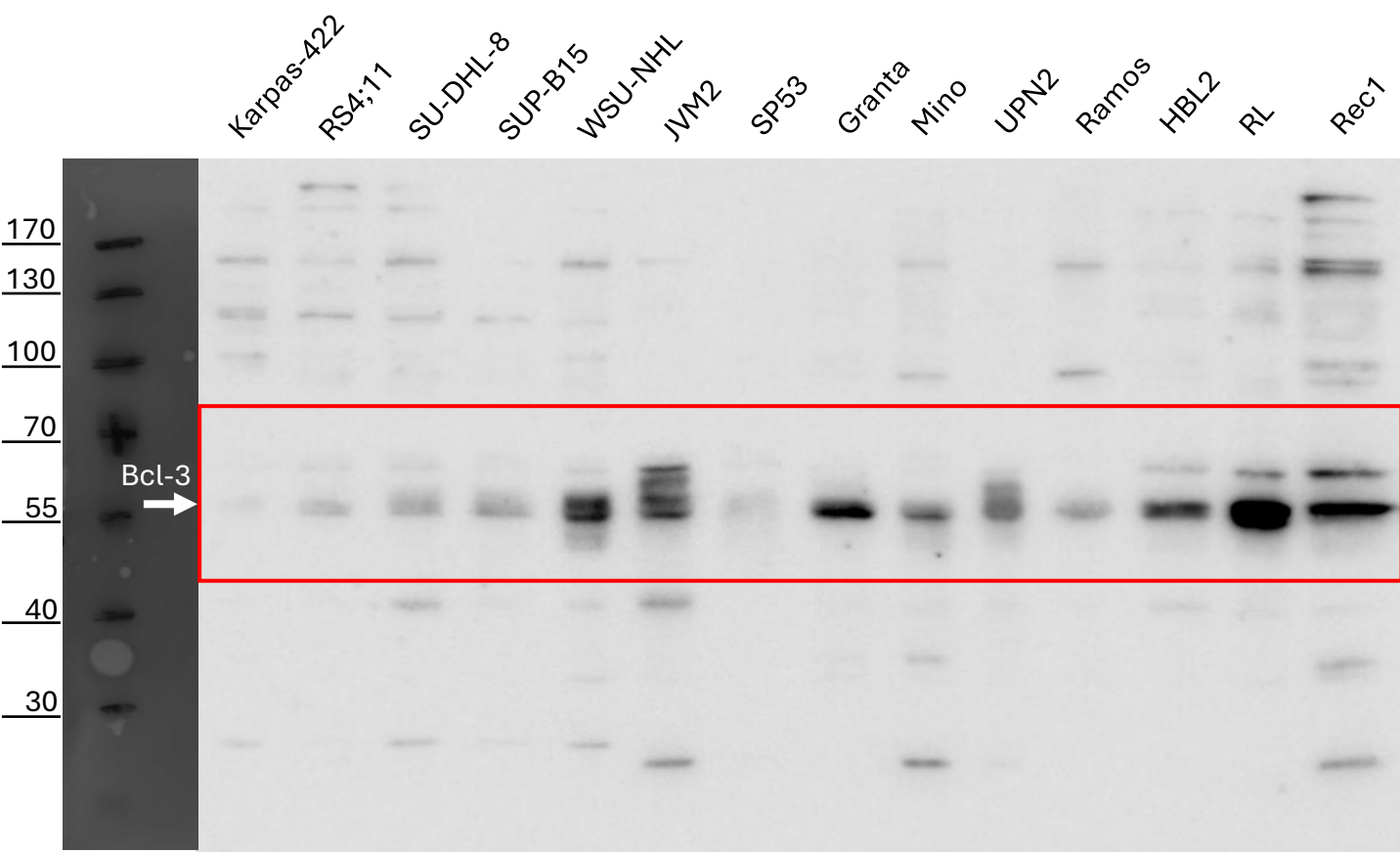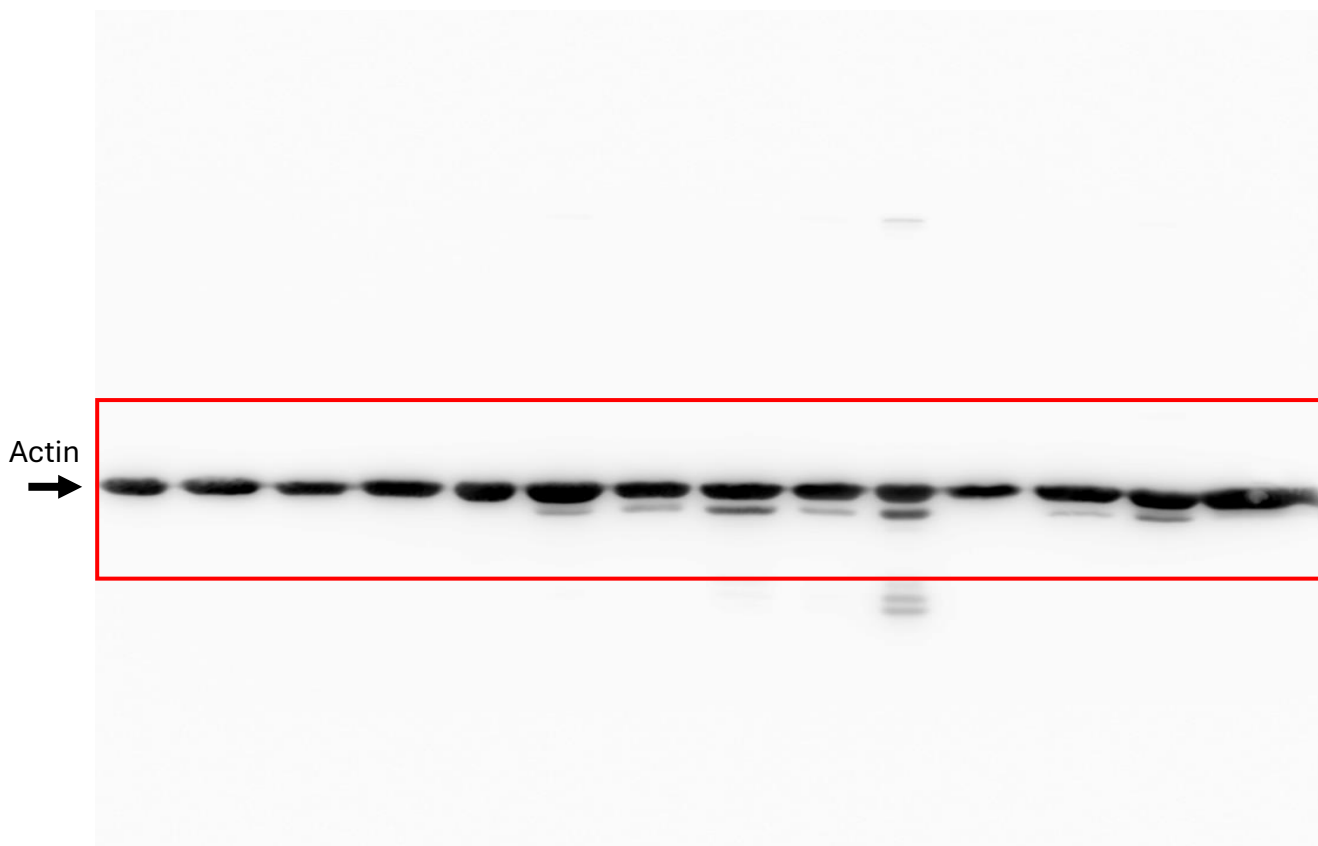

**Fig. 1D and 1E**

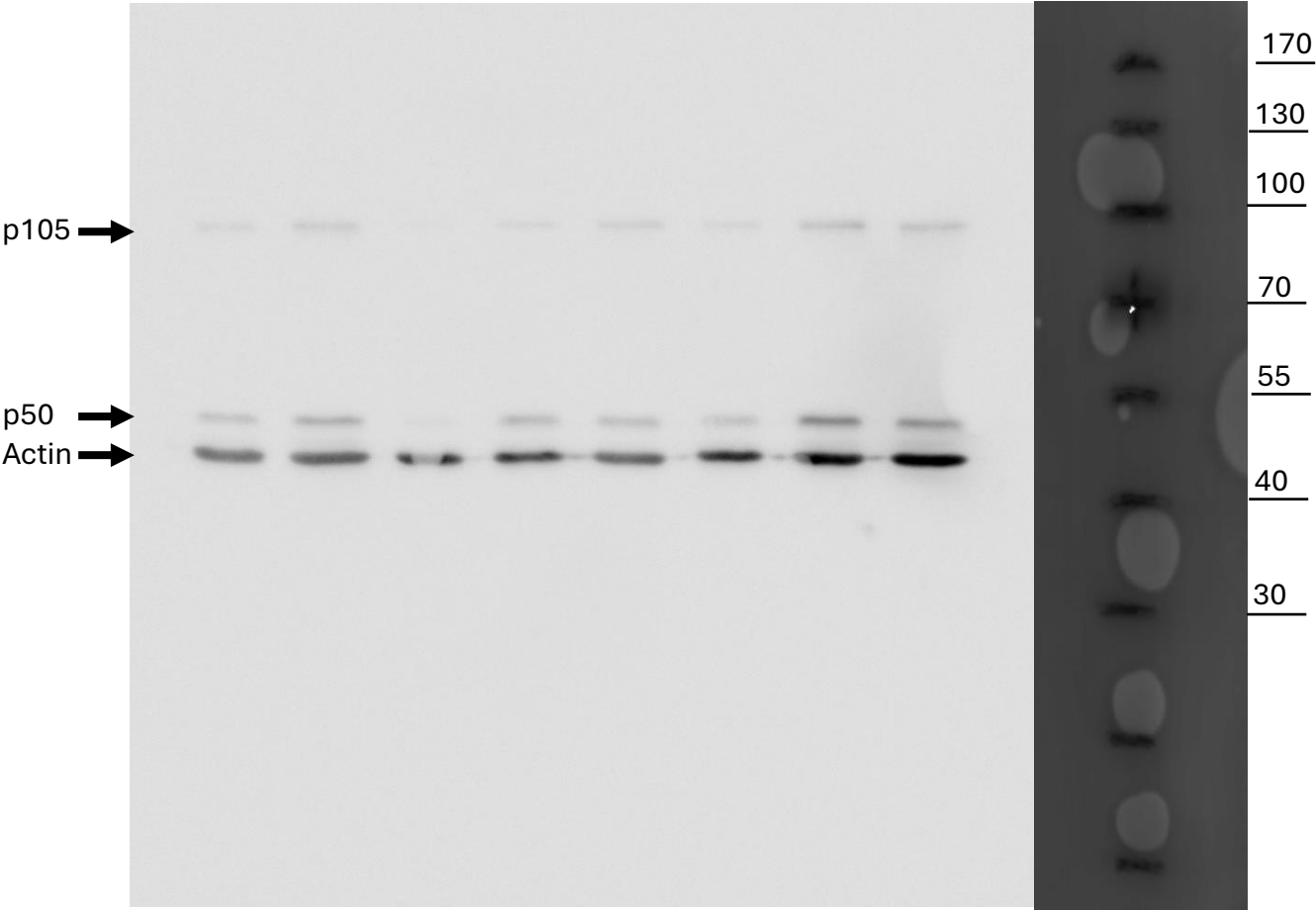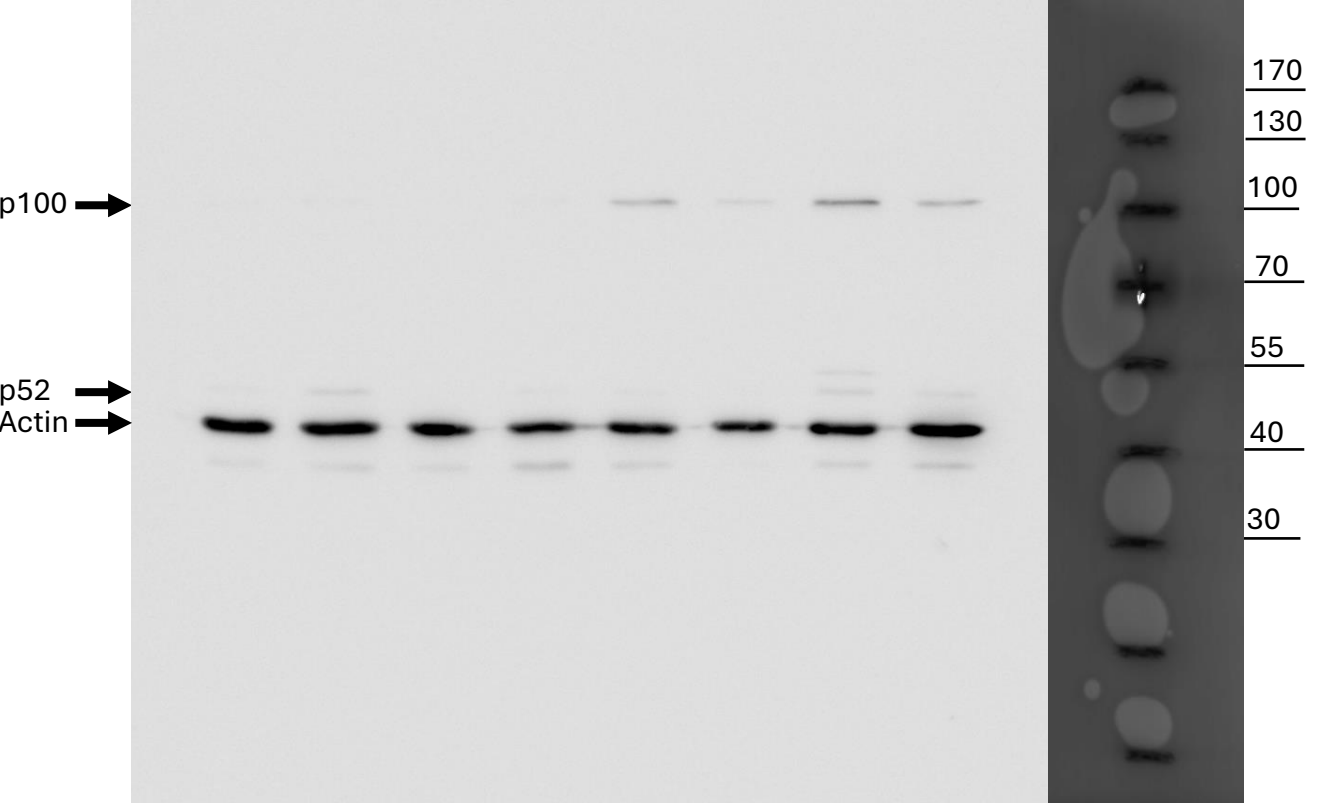

Fig. 5C

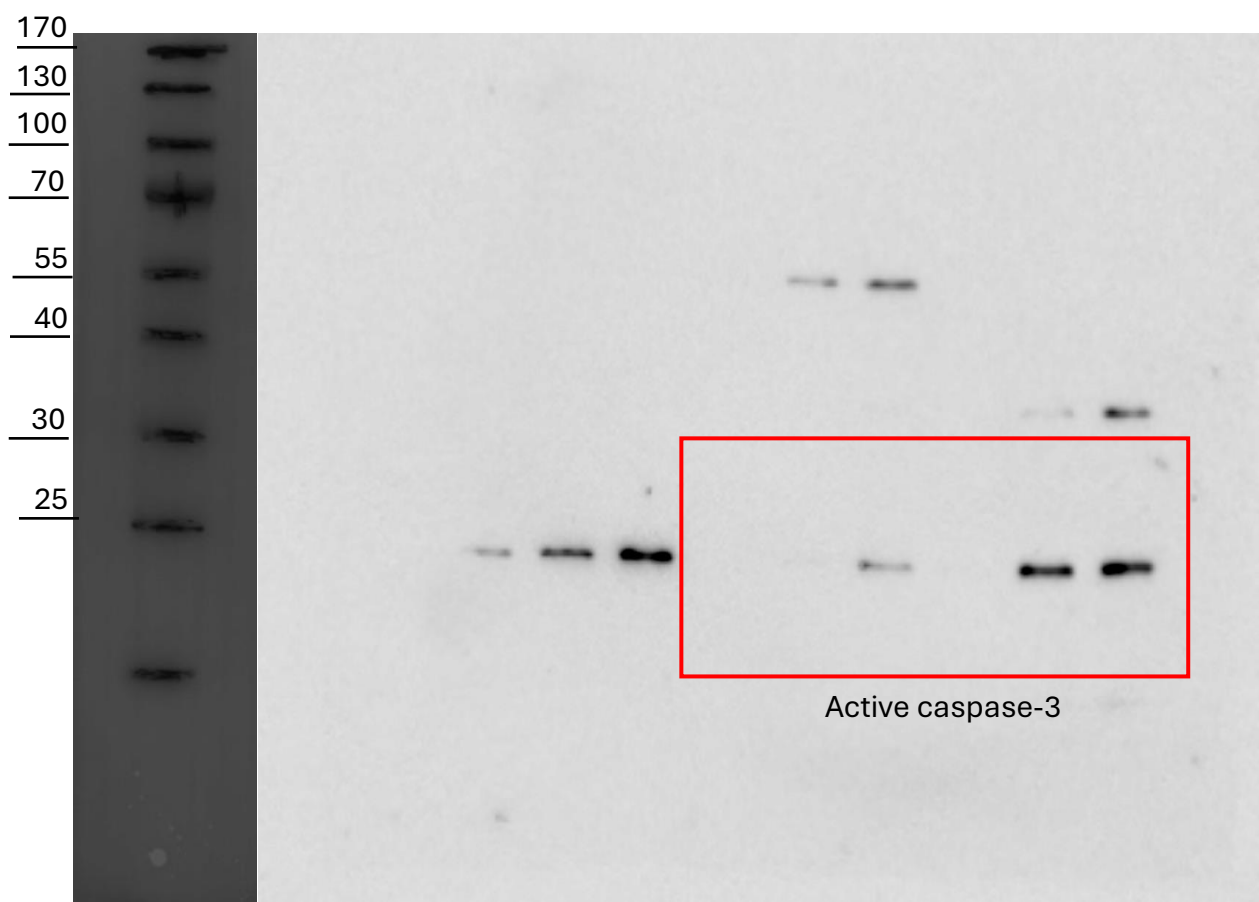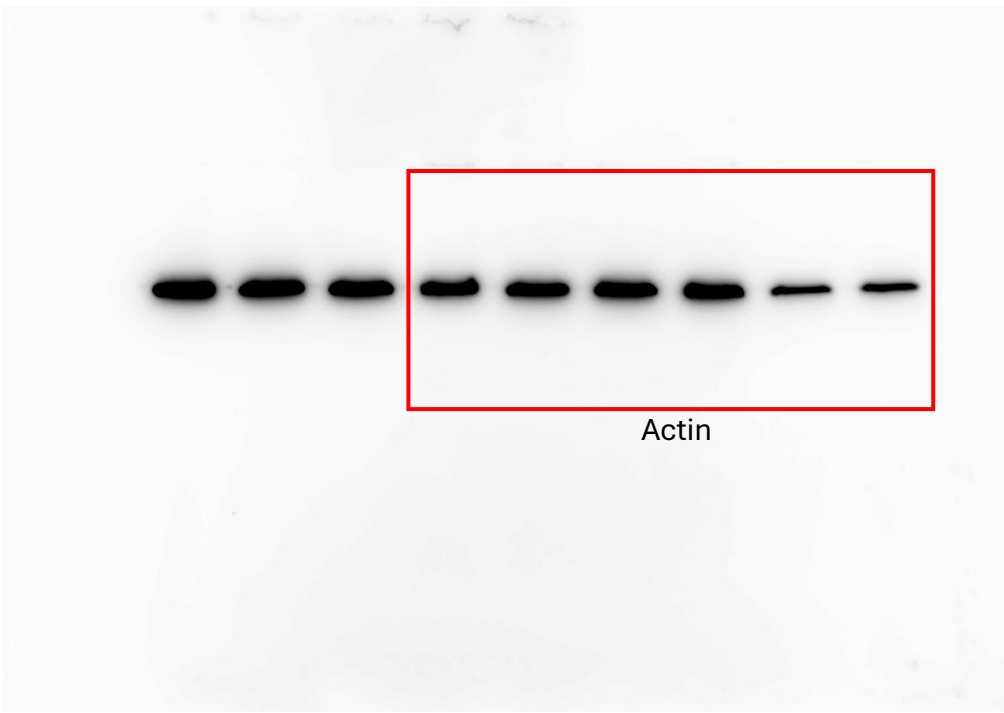

Fig. 5E

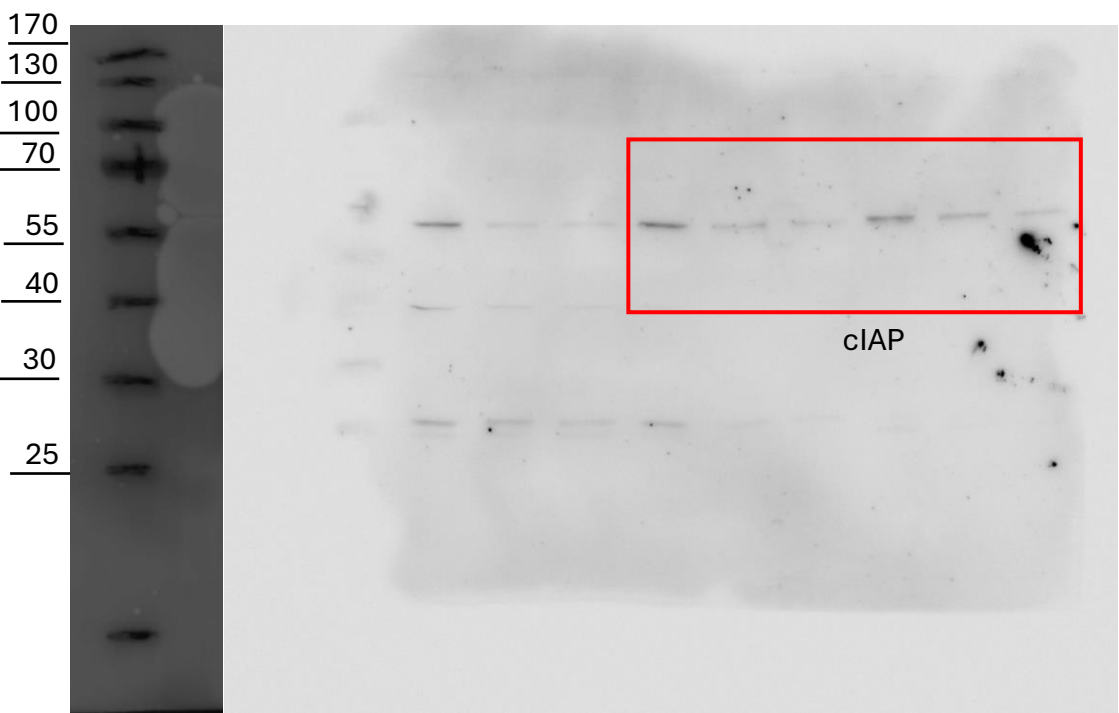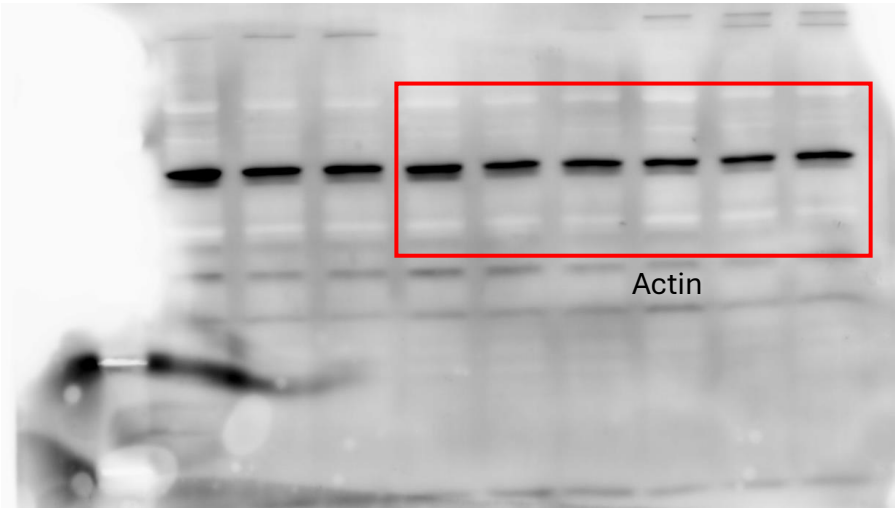

Suppl. Fig. 1B

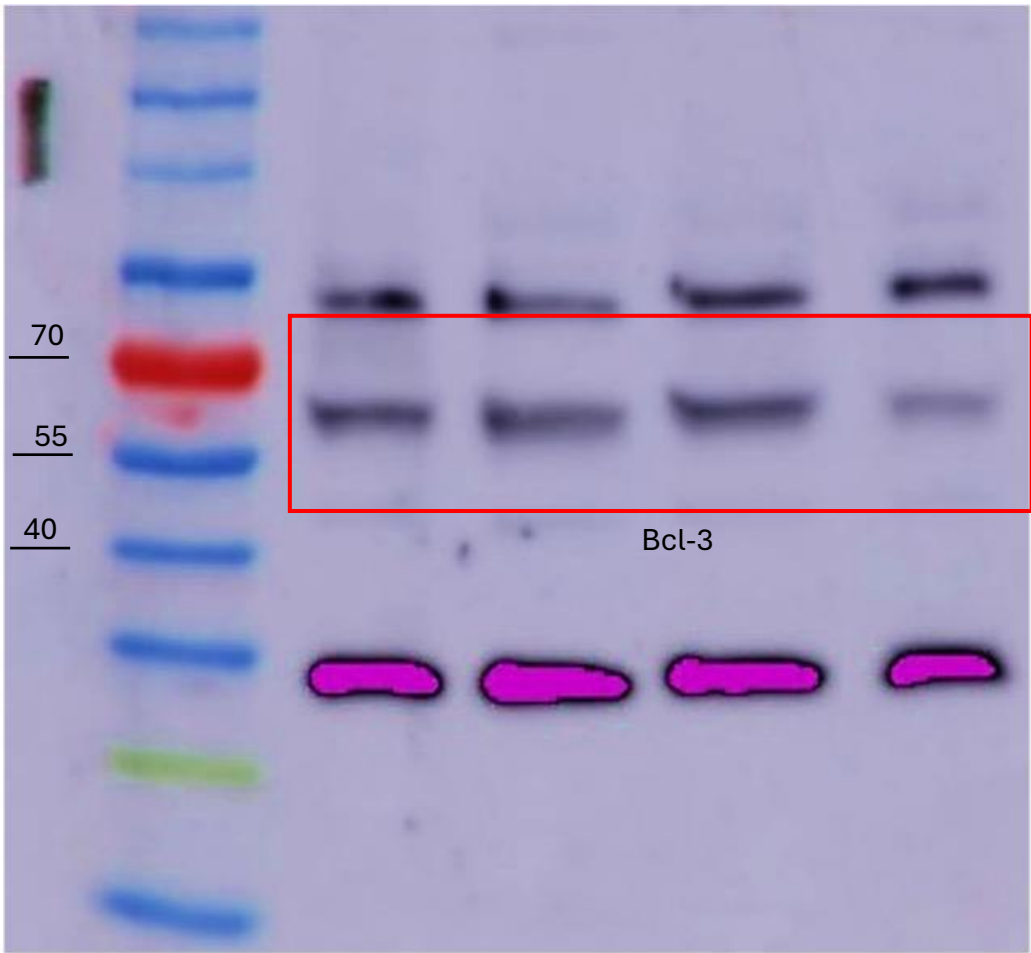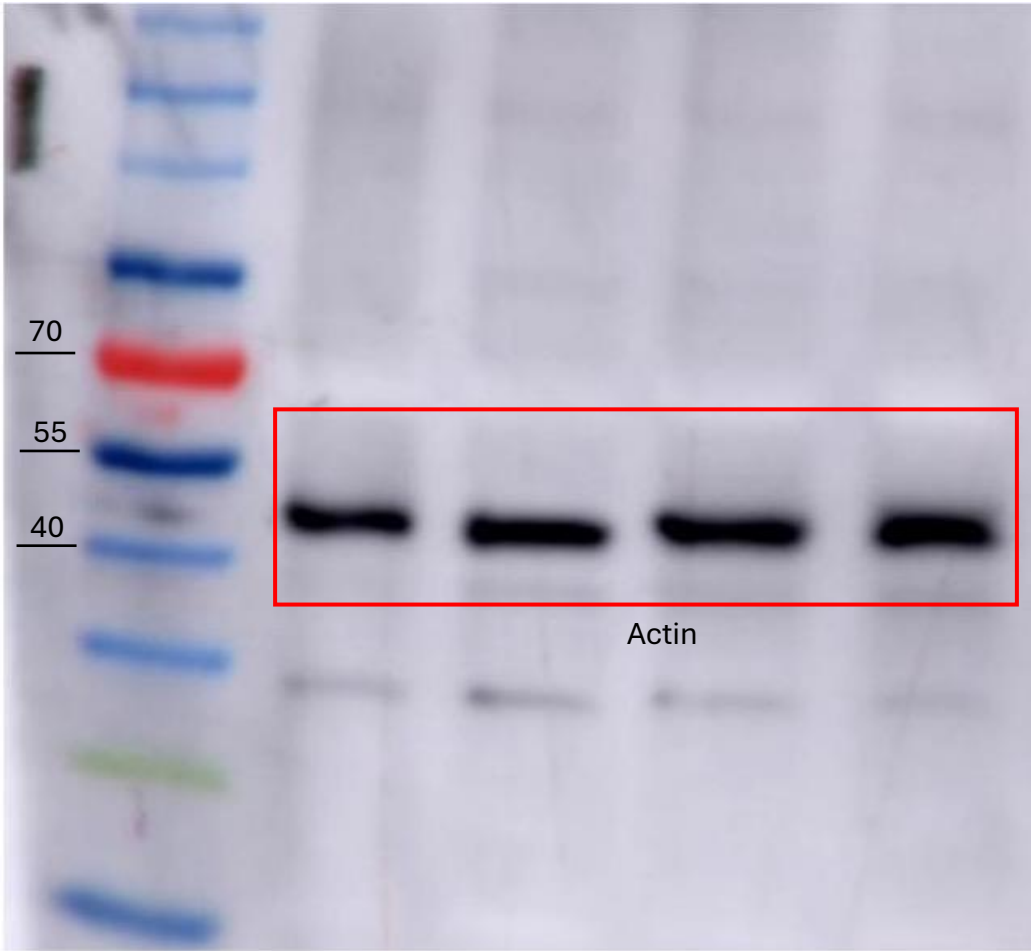

Supplement: Supplementary file 2 — Original WB data [file 41419_2024_7067_MOESM2_ESM.pdf]
